# Higher dietary flavonoid intakes are associated with lower objectively measured body composition in women: evidence from discordant monozygotic twins^1^,^2^

**DOI:** 10.3945/ajcn.116.144394

**Published:** 2017-01-18

**Authors:** Amy Jennings, Alex MacGregor, Tim Spector, Aedín Cassidy

**Affiliations:** 3 Department of Nutrition and Preventive Medicine, Norwich Medical School, University of East Anglia, Norwich, United Kingdom; and; 4 Department of Twin Research and Genetic Epidemiology, Kings College London, London, United Kingdom

**Keywords:** body composition, diet, fat distribution, flavonoids, twins

## Abstract

**Background:** Although dietary flavonoid intake has been associated with less weight gain, there are limited data on its impact on fat mass, and to our knowledge, the contribution of genetic factors to this relation has not previously been assessed.

**Objective:** We examined the associations between flavonoid intakes and fat mass.

**Design:** In a study of 2734 healthy, female twins aged 18–83 y from the TwinsUK registry, intakes of total flavonoids and 7 subclasses (flavanones, anthocyanins, flavan-3-ols, flavonols, flavones, polymers, and proanthocyanidins) were calculated with the use of food-frequency questionnaires. Measures of dual-energy X-ray absorptiometry–derived fat mass included the limb-to-trunk fat mass ratio (FMR), fat mass index, and central fat mass index.

**Results:** In cross-sectional multivariable analyses, higher intake of anthocyanins, flavonols, and proanthocyanidins were associated with a lower FMR with mean ± SE differences between extreme quintiles of −0.03 ± 0.02 (*P*-trend = 0.02), −0.03 ± 0.02 (*P*-trend = 0.03), and −0.05 ± 0.02 (*P*-trend < 0.01), respectively. These associations were not markedly changed after further adjustment for fiber and total fruit and vegetable intakes. In monozygotic, intake-discordant twin pairs, twins with higher intakes of flavan-3-ols (*n* = 154, *P* = 0.03), flavonols (*n* = 173, *P* = 0.03), and proanthocyanidins (*n* = 172, *P* < 0.01) had a significantly lower FMR than that of their co-twins with within-pair differences of 3–4%. Furthermore, in confirmatory food-based analyses, twins with higher intakes of flavonol-rich foods (onions, tea, and pears; *P* = 0.01) and proanthocyanidin-rich foods (apples and cocoa drinks; *P* = 0.04) and, in younger participants (aged <50 y) only, of anthocyanin-rich foods (berries, pears, grapes, and wine; *P* = 0.01) had a 3–9% lower FMR than that of their co-twins.

**Conclusions:** These data suggest that higher habitual intake of a number of flavonoids, including anthocyanins, flavan-3-ols, flavonols, and proanthocyanidins, are associated with lower fat mass independent of shared genetic and common environmental factors. Intervention trials are needed to further examine the effect of flavonoid-rich foods on body composition.

## INTRODUCTION

There is increasing evidence that dietary flavonoids, which are a diverse range of polyphenolic compounds that are present in plant-based foods such as fruits, vegetables, tea, wine, and chocolate, may be beneficial for weight maintenance. Higher intakes of several flavonoid subclasses including flavones, flavonols, and flavan-3-ols (catechins) have been inversely associated with BMI (in kg/m^2^) gain over 14 y (1), and pooled results from 3 prospective cohort studies in 124,086 US men and women suggested that increased intakes of most flavonoid subclasses are associated with less weight gain over 24 y, with the greatest magnitude of associations observed for anthocyanins, flavonoid polymers, and flavonols (2).

Several plausible mechanisms may link flavonoids to weight maintenance although, to date, much of the mechanistic evidence has been related to studies that were conducted with green tea extracts and their bioactive constituent, the flavan-3-ol epigallocatechin gallate (EGCG).^5^ Short-term studies in animals have provided evidence that EGCG prevents lipid absorption, decreases the expression of genes that regulate lipid metabolism, increases energy expenditure, and reduces weight gain and fat mass in a dose-dependent manner (3–7). Animal studies have also shown the effects of dietary flavanones, flavonols, and anthocyanins on obesity through mechanisms such as the inhibition of adipogenesis, the normalization of glucose tolerance, and the modulation of insulin and inflammatory signaling pathways (8–10).

Obesity, as defined by BMI, is commonly used as a proxy measure of adiposity in predicting cardiovascular and metabolic disease risk, although this definition does not differentiate between fat mass and fat-free mass or include knowledge of the pattern of fat distribution. Recently, obesity defined by the percentage of body fat was shown to be more strongly related to risk of cardiovascular disease than are obesity markers that are based on BMI or waist circumference (11). Furthermore, the body fat distribution has been shown to be a stronger determinant of insulin resistance and inflammation than is the use of body fat measurements alone (12).

Therefore, the aim of the current study was to examine, for the first time to our knowledge, the associations between intakes of the range of different flavonoid subclasses and dual-energy X-ray absorptiometry (DXA)–measured fat mass and fat mass distributions in a cohort of 2734 healthy, female twins. Furthermore, we used a discordant monozygotic twin model to examine associations between flavonoid intake and fat mass independent of genetic and shared environmental factors. On the basis of the data from previous studies and mechanistic research, we hypothesized that higher intakes of flavan-3-ols, anthocyanins, flavonoid polymers, and flavonols would be associated with lower fat mass and a more favorable fat mass distribution.

## METHODS

### Study population

Participants who were included in these analyses were female twins who were enrolled in the TwinsUK registry, which is a nationwide registry of United Kingdom adult twins who were recruited from the general population through media campaigns (13). The cohort consisted of women because, historically, the study was predominantly focused on diseases with a higher prevalence in women (osteoporosis and osteoarthritis). All participants were unaware of the specific hypotheses being tested and were not selected for particular diseases or traits. The participants have been shown to be representative of the general population in terms of disease-related characteristics and dietary intake (14, 15). The study was approved by the St. Thomas’ Hospital Research Ethics committee, and all subjects provided informed written consent.

In this study, we included 2734 female twins, aged 18–83 y, who had completed a food-frequency questionnaire (FFQ) and attended a clinical assessment for the measurement of fat mass with the use of DXA between 1996 and 2007. In total, 5772 participants completed an FFQ, of whom 17% of participants (*n* = 999) were excluded for incomplete records (on the basis of the criteria of having left >10 food items blank) or for having reported an implausible energy intake (which was defined as the ratio of energy intake to the estimated basal metabolic rate having fallen ≥2 SDs from the population mean) (**Supplemental Figure 1**).

### Assessment of fat mass

Fat mass was measured with the use of DXA according to standard protocols (QDR-2000W; Hologic) at a number of predefined anatomical regions including the trunk, arms, legs, and whole body. From these measurements, the following derivative values were calculated: the percentage of fat mass as total body fat mass (kilograms) divided by total body mass (kilograms) (multiplied by 100); the percentage central fat mass as trunk fat mass (kilograms) divided by total body mass (kilograms) (multiplied by 100); fat mass index (FMI) as fat mass (kilograms) divided by height (square meters); central fat mass index (CFMI) as trunk fat mass (kilograms) divided by height (square meters); and the fat mass ratio (FMR) as trunk fat (kilograms) divided by limb fat (kilograms). Lower values of FMR are typical of peripheral fat distribution rather than of central fat distribution. Values of FMI >9 kg/m^2^ have been shown to be comparable with BMI classifications of overweight (>25) in women (16). Because fat mass increases with greater body size, analyses for total fat mass and central fat mass variables were further adjusted for total fat-free mass (kilograms) (17).

### Assessment of flavonoid intakes

Participants completed a 131-item validated FFQ (18, 19). Flavonoid values were assigned to each of the foods that were listed in the FFQ, and for recipes, a value for each ingredient in the dishes was assigned with the use of data from the USDA as the primary data source (20, 21). For foods in the FFQ for which there were no values available in the USDA database, we searched the phenol explorer database (www.phenol-explorer.eu) to ensure that all available high-quality data on flavonoid values were included. Intakes were calculated as the frequency of each food multiplied by the nutrient content of the food for the appropriate portion size (22).

Intakes were derived for the following main subclasses of flavonoids that are habitually consumed: flavanones (eriodictyol, hesperetin, and naringenin), anthocyanins (cyanidin, delphinidin, malvidin, pelargonidin, petunidin, and peonidin), flavan-3-ols (catechins and epicatachins), flavonols (quercetin, kaempferol, myricetin, and isohamnetin), flavones (luteolin and apigenin), polymers [including proanthocyanidins (excluding monomers), theaflavins, and thearubigins], and proanthocyanidins (dimers, trimers, 4–6 mers, 7–10 mers, polymers, and monomers). Total flavonoid intakes were derived from the addition of 6 component subclasses (flavanones, anthocyanins, flavan-3-ols, flavonols, flavones, and polymers). Proanthocyanidins were included in the polymer and as monomers in the flavan-3-ol subclasses. We also calculated the main dietary sources that contributed to intakes of the different flavonoid subclasses and identified foods that contributed ≥10% of intake. Mean intakes of the various flavonoid subclasses were shown to be similar to those previously reported in the European Prospective Investigation into Cancer and Nutrition United Kingdom general population cohort (23–25).

### Assessment of covariates

Intakes of energy and other nutrients were determined from the FFQ that previously described, with the use of values from the United Kingdom, national food-composition tables (26). Information on smoking, medication use, and menopausal status was obtained with the use of a standardized nurse-administered questionnaire. Participants completed a questionnaire that detailed their self-reported physical activity levels during leisure time and at work during the past 12 mo, whereby 1 indicated inactive, 2 indicated light activity, 3 indicated moderate activity, and 4 indicated heavy activity (27). These classifications have shown to be significantly correlated with a more in-depth assessment of recordings of how much time subjects spent in moderate and vigorous non–weight-bearing and weight-bearing activity per week. For participants who completed the more in-depth assessments, the mean time spent in leisure-time physical activity per week for each physical activity level was estimated as follows: inactive, 16 min; light activity, 36 min; moderate activity, 102 min; and heavy activity, 199 min (27). Height was measured to the nearest 0.5 cm with the use of a wall-mounted stadiometer. Zygosity was ascertained with the use of a questionnaire and confirmed via the subsequent genotyping as part of genome-wide association studies (PE Applied Biosystems).

### Statistical analysis

First, we used all participants and treated twins as individuals (individual-level analysis) while accounting for twin-pair clustering. Participants were ranked into quintiles of intake for both flavonoid subclasses and foods that contributed significantly to intake of each subclass (>10%). Associations with fat mass variables were assessed with the use of an ANCOVA (*n* = 2734, *n* = 1207 twin-pairs, and *n* = 320 individuals). We present our data as absolute differences in fat mass variables between extreme quintiles (quintile 5 minus quintile 1) or as the percentage difference between extreme quintiles (quintile 5 minus quintile 1, divided by quintile 1, and multiplied by 100). All models were adjusted for age (years), current smoking (yes or no), physical activity (inactive, moderately active, and active), menopausal status (premenopausal or postmenopausal), use of hormone replacement therapy (yes or no), use of vitamin supplements (yes or no), alcohol consumption (rarely or never, <1 drink/mo, or ≥1 drink/mo), and intakes of energy (kilocalories per day in quintiles), caffeine (micrograms per day in quintiles), saturated fat (grams per day in quintiles), polyunsaturated fat (grams per day in quintiles), monounsaturated fat (grams per day in quintiles), and sugar-sweetened beverages (grams per day in quartiles). In secondary analyses, we further adjusted flavonoid-subclass models for fiber intake and fruit and vegetable intake both individually and in combination. In a sensitivity analysis, we performed multiple imputations of missing data to check for selection bias with the use of Markov-chain Monte Carlo methods (28). We imputed missing values for participants who completed an eligible FFQ but who did not attend for a DXA scan (*n* = 2039). Our imputation model included all variables from our analysis model as well as body weight. We performed 45 imputations (equal to the percentage of incomplete cases) (29). Similarly, we imputed values for missing data for participants who completed an FFQ with the inclusion of subjects who were excluded for reporting implausible energy intakes but did not attend for a DXA scan (*n* = 2812) with the use of the same methods. Because the observed estimates for our primary outcome measure (FMR) were similar in the original and imputed data sets, we only present the results of the analysis in the complete data set.

Finally, we compared our primary outcome measure (FMR) in monozygotic co-twins who were discordant for intakes of the flavonoid subclasses and main foods sources of each subclass that were significantly associated with body fat in our cross-sectional analyses. Discordance was defined as a within-pair difference in intake of ≥1 SD. We assigned each twin within a discordant pair to higher or lower intake for each subclass, and with the use of a paired sample *t* tests, we examined whether the FMR differed between the twin with higher intake and that of the co-twin with lower intake. To eliminate other known environmental influences on the FMR, we ensured there were no significant differences between higher and lower intake pairs for smoking status, physical activity, menopausal status, use of hormone replacement therapy, and alcohol consumption with the use of a McNemar chi-square test. Because the FMR is known to be strongly related to age and menopausal status, we repeated the co-twin analyses with participants aged <50 y (30, 31). A post hoc power calculation that was conducted for the FMR in monozygotic discordant twins that was based on our findings showed >99% power (2 tailed; α = 0.05) to detect a mean ± SE difference of 0.04 ± 0.01 in the FMR in 172 twin pairs (calculated with the use of G*Power version 3.1, Heinrich Heine University).

*P* < 0.05 was considered statistically significant for all analyses. Statistical analyses were performed with Stata statistical software (version 11; StataCorp LP).

## RESULTS

Characteristics and dietary intakes of the 2734 female participants, aged 18–83 y, are shown in **Table 1**; 43% of subjects (*n* = 1174) were monozygotic twins. Median total flavonoid intake was 1.1 g/d (IQR: 0.5–1.7 g/d), and polymers and flavan-3-ols made the greatest contributions to intake (68% and 19%, respectively). In total, 38% of participants (*n* = 1135) had FMI values >9 kg/m^2^.

**TABLE 1:** Characteristics and dietary intake of the 2734 female twins

	Value
Age, y	53.0 (45, 60)^1^
Zygosity, monozygotic, % (*n*)	43 (1174)
Current smoking, yes, % (*n*)	15 (412)
Physically active, yes, % (*n*)	25 (671)
Postmenopausal, yes, % (*n*)	63 (1729)
Hormone replacement therapy use, yes, % (*n*)	15 (413)
Vitamin supplement use, yes, % (*n*)	54 (1487)
Alcohol use, ≥1 drink/mo, % (*n*)	83 (2269)
Fat mass ratio, kg	0.9 (0.7, 1.1)
Fat mass index, kg/m^2^	8.1 (6.4, 10.3)
Central fat mass index, kg/m^2^	3.9 (2.8, 5.2)
Total body fat, %	36.1 (31.1, 40.8)
Central body fat, %	33.1 (26.8, 38.7)
Total flavonoids, mg/d	1082 (542, 1674)
Flavanones, mg/d	20.6 (8.7, 43.2)
Anthocyanins, mg/d	16.9 (9.5, 27.7)
Flavan-3-ols, mg/d	212 (91.9, 351)
Flavonols, mg/d	44.0 (29.1, 61.2)
Flavones, mg/d	1.9 (1.1, 2.9)
Polymers, mg/d	761 (355, 1209)
Proanthocyanidins, mg/d	257 (186, 334)
Caffeine, mg/d	252 (154, 340)
Energy, kcal/d	1869 (1537, 2255)
Saturated fat, g/d	23.2 (17.4, 30.5)
Monounsaturated fat, g/d	21.1 (16.2, 27.1)
Polyunsaturated fat, g/d	14.4 (11.0, 18.7)
Sugar-sweetened beverages, g/d	11.4 (0, 68.6)
Fiber, g/d	19.4 (15, 24.7)
Fruit and vegetables, g/d	501 (338, 680)

1 Median; IQR in parentheses (all such values).

Higher intakes of anthocyanin, flavonol, and proanthocyanidin subclasses were significantly associated with a more favorable fat mass distribution, which was defined according to the ratio of limb fat mass to trunk fat mass (**Table 2**). The magnitude of these associations was 4–5% for the FMR for the comparison of extreme quintiles of intake [anthocyanins (mean ± SE of quintile 5 − quintile 1): −0.03 ± 0.02 kg:kg (*P*-trend = 0.02); flavonols (quintile 5 − quintile 1): −0.03 ± 0.02 kg:kg (*P*-trend = 0.03); and proanthocyanidins (quintile 5 − quintile 1): −0.05 ± 0.02 kg:kg (*P*-trend < 0.01)]. Age, the use of vitamin supplements, and intakes of monounsaturated fat and whole grains were the only covariates that were significantly associated with the FMR in these models (for all flavonoid subclasses).

**TABLE 2:** Fat mass and fat mass distribution by quintiles of flavonoid subclass intake in 2734 women aged 18–83 y^1^

Flavonoid subclass	FMR, kg:kg	Central FM, %	CFMI, kg/m^2^	FM, %	FMI, kg/m^2^
Total flavonoids, mg/d					
Quintile 1	0.93 ± 0.01	33.2 ± 0.4	4.3 ± 0.1	36.2 ± 0.3	8.8 ± 0.1
Quintile 2	0.92 ± 0.01	33.0 ± 0.4	4.2 ± 0.1	36.1 ± 0.3	8.8 ± 0.1
Quintile 3	0.89 ± 0.01	31.9 ± 0.4	4.0 ± 0.1	35.4 ± 0.3	8.4 ± 0.1
Quintile 4	0.91 ± 0.01	32.7 ± 0.4	4.1 ± 0.1	35.9 ± 0.3	8.7 ± 0.1
Quintile 5	0.90 ± 0.01	32.0 ± 0.4	4.0 ± 0.1	35.4 ± 0.3	8.5 ± 0.1
* P*	0.06	0.08	0.05	0.15	0.13
* P*^2^	0.06	0.10	0.06	0.16	0.13
Flavanones, mg/d					
Quintile 1	0.91 ± 0.01	33.0 ± 0.3	4.2 ± 0.1	36.3 ± 0.3	8.8 ± 0.1
Quintile 2	0.91 ± 0.01	33.1 ± 0.3	4.2 ± 0.1	36.1 ± 0.3	8.8 ± 0.1
Quintile 3	0.89 ± 0.01	32.3 ± 0.3	4.1 ± 0.1	35.7 ± 0.3	8.6 ± 0.1
Quintile 4	0.90 ± 0.01	32.0 ± 0.3	4.0 ± 0.1	35.3 ± 0.3	8.4 ± 0.1
Quintile 5	0.92 ± 0.01	32.5 ± 0.4	4.1 ± 0.1	35.6 ± 0.3	8.5 ± 0.1
* P*	0.81	0.04	0.04	0.02	0.01
* P*^2^	0.59	0.09	0.06	0.03	0.01
Anthocyanins, mg/d					
Quintile 1	0.92 ± 0.01	33.3 ± 0.4	4.3 ± 0.1	36.3 ± 0.3	8.8 ± 0.1
Quintile 2	0.92 ± 0.01	32.6 ± 0.3	4.1 ± 0.1	35.8 ± 0.3	8.6 ± 0.1
Quintile 3	0.90 ± 0.01	32.3 ± 0.3	4.1 ± 0.1	35.6 ± 0.3	8.5 ± 0.1
Quintile 4	0.90 ± 0.01	32.6 ± 0.4	4.1 ± 0.1	35.9 ± 0.3	8.6 ± 0.1
Quintile 5	0.89 ± 0.01	32.1 ± 0.4	4.0 ± 0.1	35.5 ± 0.3	8.5 ± 0.1
* P*	0.02	0.01	0.04	0.21	0.21
* P*^2^	0.02	0.03	0.07	0.33	0.23
Flavan-3-ols, mg/d					
Quintile 1	0.92 ± 0.01	33.2 ± 0.4	4.3 ± 0.1	36.4 ± 0.3	8.9 ± 0.1
Quintile 2	0.91 ± 0.01	33.0 ± 0.4	4.2 ± 0.1	36.1 ± 0.3	8.8 ± 0.1
Quintile 3	0.89 ± 0.01	32.0 ± 0.4	4.0 ± 0.1	35.4 ± 0.3	8.4 ± 0.1
Quintile 4	0.90 ± 0.01	32.8 ± 0.3	4.2 ± 0.1	36.0 ± 0.3	8.7 ± 0.1
Quintile 5	0.90 ± 0.01	31.8 ± 0.4	4.0 ± 0.1	35.2 ± 0.3	8.4 ± 0.1
* P*	0.08	0.03	0.02	0.05	0.04
* P*^2^	0.09	0.04	0.02	0.05	0.04
Flavonols, mg/d					
Quintile 1	0.93 ± 0.01	33.2 ± 0.4	4.3 ± 0.1	36.2 ± 0.3	8.8 ± 0.1
Quintile 2	0.90 ± 0.01	32.8 ± 0.4	4.2 ± 0.1	36.1 ± 0.3	8.8 ± 0.1
Quintile 3	0.91 ± 0.01	32.5 ± 0.4	4.1 ± 0.1	35.7 ± 0.3	8.5 ± 0.1
Quintile 4	0.90 ± 0.01	32.6 ± 0.3	4.1 ± 0.1	35.9 ± 0.3	8.7 ± 0.1
Quintile 5	0.89 ± 0.01	31.8 ± 0.4	3.9 ± 0.1	35.1 ± 0.3	8.3 ± 0.1
* P*	0.03	0.02	0.01	0.04	0.03
* P*^2^	0.04	0.02	0.01	0.04	0.03
Flavones, mg/d					
Quintile 1	0.92 ± 0.01	33.6 ± 0.4	4.4 ± 0.1	36.7 ± 0.3	9.0 ± 0.1
Quintile 2	0.90 ± 0.01	32.8 ± 0.3	4.2 ± 0.1	36.2 ± 0.3	8.7 ± 0.1
Quintile 3	0.91 ± 0.01	32.4 ± 0.3	4.1 ± 0.1	35.7 ± 0.3	8.6 ± 0.1
Quintile 4	0.89 ± 0.01	32.0 ± 0.4	4.0 ± 0.1	35.5 ± 0.3	8.5 ± 0.1
Quintile 5	0.92 ± 0.01	32.0 ± 0.4	4.0 ± 0.1	35.1 ± 0.3	8.3 ± 0.1
* P*	0.64	<0.01	<0.01	<0.01	<0.01
* P*^2^	0.86	<0.01	<0.01	<0.01	<0.01
Polymers, mg/d					
Quintile 1	0.93 ± 0.01	33.0 ± 0.4	4.2 ± 0.1	36.1 ± 0.3	8.8 ± 0.1
Quintile 2	0.92 ± 0.01	33.1 ± 0.4	4.2 ± 0.1	36.1 ± 0.3	8.8 ± 0.1
Quintile 3	0.89 ± 0.01	31.9 ± 0.4	4.0 ± 0.1	35.3 ± 0.3	8.4 ± 0.1
Quintile 4	0.91 ± 0.01	32.9 ± 0.3	4.2 ± 0.1	36.1 ± 0.3	8.8 ± 0.1
Quintile 5	0.90 ± 0.01	32.0 ± 0.4	4.0 ± 0.1	35.4 ± 0.3	8.5 ± 0.1
* P*	0.05	0.15	0.10	0.31	0.24
* P*^2^	0.05	0.18	0.11	0.32	0.24
Proanthocyanidins, mg/d					
Quintile 1	0.95 ± 0.01	34.0 ± 0.3	4.2 ± 0.1	36.8 ± 0.3	9.0 ± 0.1
Quintile 2	0.92 ± 0.01	32.9 ± 0.3	4.2 ± 0.1	36.0 ± 0.3	8.7 ± 0.1
Quintile 3	0.89 ± 0.01	32.4 ± 0.3	4.0 ± 0.1	35.8 ± 0.3	8.6 ± 0.1
Quintile 4	0.89 ± 0.01	32.1 ± 0.3	4.2 ± 0.1	35.5 ± 0.3	8.4 ± 0.1
Quintile 5	0.89 ± 0.01	31.6 ± 0.4	4.0 ± 0.1	35.0 ± 0.3	8.3 ± 0.1
* P*	<0.01	<0.01	<0.01	<0.01	<0.01
* P*^2^	<0.01	<0.01	<0.01	<0.01	<0.01

1 All values are adjusted means ± SEs. *n* = 2734. Means were adjusted for age, smoking, physical activity, menopausal status, use of hormone replacement therapy, vitamin-supplement use, alcohol use, and intakes of energy, caffeine, saturated fat, polyunsaturated fat, monounsaturated fat, whole grains, and sugar-sweetened beverages. *P* values are for trends that were calculated with the use of an ANCOVA. CFMI, central fat mass index; FM, fat mass; FMI, fat mass index; FMR, fat mass ratio.

2 Further adjusted for fruit and vegetable intake and fiber intake.

Increased intake of all flavonoid subclasses, with the exception of polymers, were significantly associated with lower central fat mass either when expressed as a percentage of the total mass or when adjusted for height as the CFMI (Table 2). The strongest inverse associations were observed for flavonol, flavone, and proanthocyanidin subclasses. For proanthocyanidins, there was an absolute difference in the mean ± SE percentage of central fat mass of −2.4% ± 0.5% (*P*-trend < 0.01) and in CFMI of −0.5 ± 0.1 kg/m^2^ (*P*-trend < 0.01) for the comparison of extreme quintiles of intake. For FMI, inverse associations ranged from a mean ± SE of −0.3 ± 0.2 kg/m^2^ (*P*-trend = 0.01) for flavanones to −0.7 ± 0.2 kg/m^2^ (*P*-trend < 0.01) for proanthocyanidins for the comparison of lowest and highest intakes with no associations observed for intake of anthocyanins or polymers.

Our results were not markedly changed after the addition of intake of fiber or fruit and vegetables, either individually or when added together to our final multivariate model. For example, when both fiber and fruit and vegetable intakes were added to the multivariable model, differences in fat mass variables between extreme quintiles of intake were −4.3% for the FMR and anthocyanin intake (mean ± SE of quintile 5 − quintile 1: −0.04 ± 0.02 kg:kg; *P*-trend = 0.02) and −0.49 kg/m^2^ for the CFMI and proanthocyanidin intake (quintile 5 − quintile 1: −0.49 ± 0.11 kg/m^2^; *P*-trend < 0.01).

In sensitivity analyses, we tested the primary outcome measure FMR after imputation of missing values. In our complete case analysis, the mean ± SE coefficient for the FMR per quintile of anthocyanin intake was −0.01 ± 0.004 kg:kg (*P*-trend = 0.02) compared with −0.01 ± 0.003 kg:kg (*P*-trend = 0.04) in analysis with missing data imputed. Likewise, for proanthocyanidins, the mean coefficient for the FMR was −0.01 kg:kg in both the complete case analysis (±0.004; *P*-trend < 0.01) and imputed analysis (±0.003; *P*-trend = 0.05).

**FIGURE 1:**
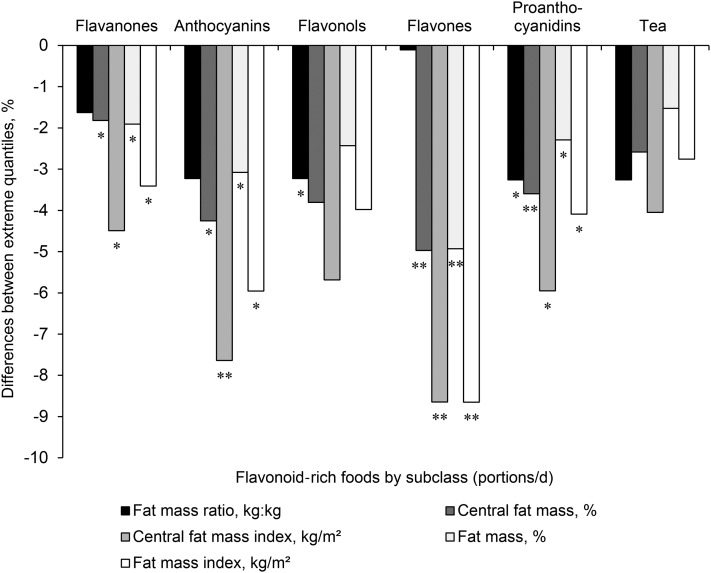
Percentage differences in fat mass and fat mass distribution between extreme quantiles of intake of flavonoid-rich foods in 2734 women aged 18–83 y. Bars represent percentage differences in outcome measures between extreme quantiles of intake (portions per day). Quantile limits were selected on the basis of the best data distribution. Flavanone-rich foods (quintiles) were oranges (120 g), grapefruit (80 g), and fruit juice (160 g); anthocyanin-rich foods (quintiles) were berries (100 g), pears (170 g), grapes (80 g), and wine (125 mL); flavonol-rich foods (quintiles) were pears (170 g), tea (260 mL), and onions (60 g); flavone-rich foods (quintiles) were wine (125 mL), oranges (120 g), and peppers (80 g); and proanthocyanidin-rich foods (tertiles) were apples (100 g) and a cocoa beverage with milk (260 mL). Tea (260 mL) was the only food that contributed ≥10% to intakes of total flavonoids, flavan-3-ols, and polymers and was included individually in the figure (quartiles). *^,^**P values were calculated with the use of an ANCOVA with age, smoking, physical activity, menopausal status, hormone replacement therapy use, vitamin-supplement use, alcohol use, and intakes of energy, caffeine, saturated fat, polyunsaturated fat, monounsaturated fat, whole grains, and sugar-sweetened beverages as covariates: *P < 0.05, **P < 0.01.

In confirmatory food-based analyses, higher pooled intake of foods rich in flavanones (citrus fruit; oranges, grapefruit, and fruit juice), anthocyanins (berries, pears, grapes, and wine), flavones (oranges, peppers, and wine), and proanthocyanidins (apples and cocoa drinks) were inversely associated with significantly lower fat mass and central fat mass (**Figure 1**). The greatest magnitudes of association were for the foods that contributed to the anthocyanin and flavone subclasses with higher intakes that were related to 6–9%-lower FMI and 8–9%-lower CFMI for the comparison of extreme quintiles of intake. These findings related to a 2.6-portion/d difference in anthocyanin intake and a 2.7-portion/d difference in flavone intake. One portion of anthocyanin-rich foods equated to 100 g berries, 170 g pears, 80 g grapes, or 125 mL wine, and 1 portion of flavone-rich foods equated to 125 mL wine, 120 g oranges, or 80 g peppers. Although tea was the only significant contributor (>10%) to intakes of flavan-3-ols and polymers, intake was not significantly associated with any of the fat mass outcomes assessed.

**FIGURE 2:**
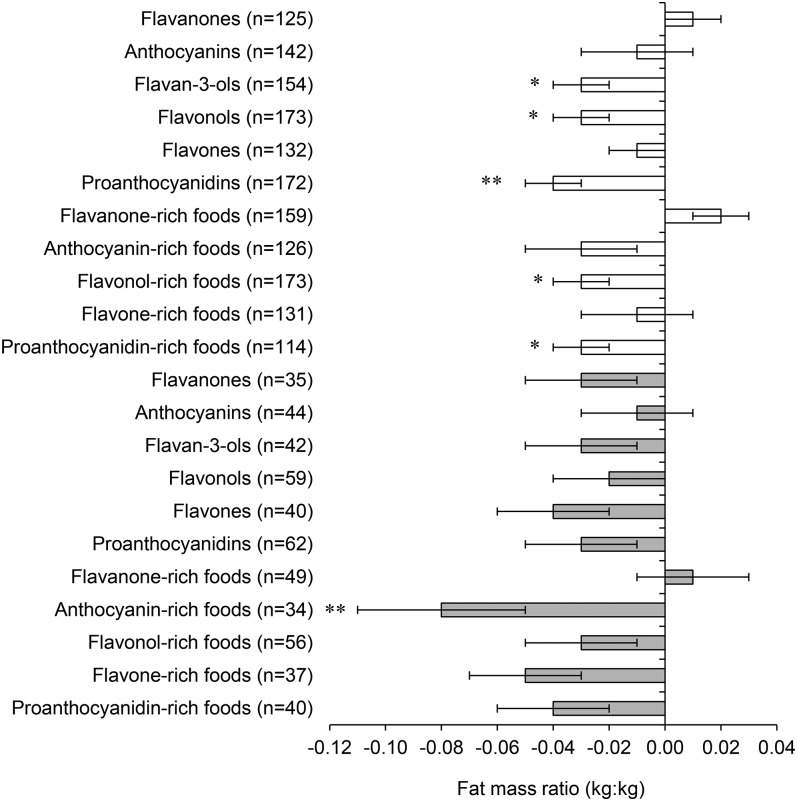
Mean ± SE differences in the fat mass ratio between monozygotic co-twins who were discordant for intakes of different flavonoid subclasses. Bars represent differences between twins with higher intake and twins with lower intake for the whole cohort (open bars) and in twins <50 y old (shaded bars). Discordance was defined as a within-pair difference in intake ≥1 SD. Flavanone-rich foods were oranges (120 g), grapefruit (80 g) and fruit juice (160 g); anthocyanin-rich foods were berries (100 g), pears (170 g), grapes (80 g), and wine (125 mL); flavonol-rich foods were pears (170 g), tea (260 mL), and onions (60 g); flavone-rich foods were wine (125 mL), oranges (120 g), and peppers (80 g); and proanthocyanidin-rich foods were apples (100 g) and a cocoa beverage with milk (260 mL). *^,^**For comparisons of twins with higher intake with twins with lower intake (paired sample *t* tests): *P < 0.05, **P < 0.01.

In our co-twin case-control analyses, we examined monozygotic twin pairs who were discordant for intakes of the different flavonoid subclasses and for the main contributors to habitual intakes. Within each twin pair, the twin with higher intakes of flavan-3-ols (*P* = 0.03), flavonols (*P* = 0.03), and proanthocyanidins (*P* < 0.01) had a significantly lower mean ± SE FMR (proanthocyanidins: −0.04 ± 0.01; all other subclasses: −0.03 ± 0.01) (**Figure 2**). Intakes between monozygotic twin pairs differed by 308 mg for flavan-3-ols, 34.8 mg for flavonols, and 198 mg for proanthocyanidins. We also showed a significantly lower mean ± SE FMR after higher intake of foods that were rich in flavonols (−0.03 ± 0.01; *P* = 0.01) and proanthocyanidins (−0.03 ± 0.01; *P* = 0.04). Because the FMR is known to be strongly related to age and menopausal status, we repeated our co-twin analyses but restricted the analysis to participants aged <50 y. In these younger participants, we further observed a significant difference in the FMR of 9% (mean ± SE: −0.08 ± 0.03; *P* = 0.01) for the comparison of monozygotic co-twins with high intake of anthocyanin-rich foods compared with monozygotic co-twins with low intake of anthocyanin-rich foods. These amounts equated to a mean difference in intake of 2 portions anthocyanin-rich foods/d (berries, pears, grapes, and wine).

## DISCUSSION

To our knowledge, this is the first study to have examined the associations between flavonoid subclasses and objectively DXA-measured fat mass and fat mass distribution. It was a particular strength of these analyses that we were able to control for potential genetic confounding with the use of a cohort of carefully phenotyped twins who were discordant for flavonoid intake. We showed, in cross-sectional analyses of 2734 women, that higher intakes of anthocyanins and flavonols were associated with lower fat mass and reduced central adiposity. These inverse associations were independent of established dietary and other risk factors, including physical activity, which have previously been associated with fat mass. Even the addition of total fruit and vegetable intake and fiber intake to our model did not substantially attenuate the relation, which suggested that the observed effects were specific to a food constituent in these flavonoid-rich foods and not necessarily related to participants who ate high habitual intakes of fruit and vegetables. When we further explored these relations in intake-discordant monozygotic twins, we showed that twins with higher intakes of flavan-3-ols (308 mg), flavonols (34.8 mg), and proanthocyanidins (198 mg) and, in younger (<50 y old) twin-pairs, of anthocyanin-rich foods (2 portions), had significantly a lower FMR than that of their co-twins. There were differences in the FMR (trunk mass:limb fat mass) of 0.03–0.08 kg or 3–9% for each subclass.

The body fat distribution may be expressed as trunk fat divided by leg fat or trunk fat divided by limb fat (16). Higher values of trunk fat and leg fat are associated with increased prevalence of adverse health outcomes independent of total and regional fat distributions (30). Specifically, a comparison of extreme quartiles of trunk-to-leg volume in the NHANES data set (quintile 1, <1.34; quintile 4, ≥1.66), there was a reported difference in the prevalence of diabetes of 20%, of high triglycerides of 40%, of low HDL of 23%, of high blood pressure of 28%, and of metabolic syndrome of 25% (30).

The scale of our associations for the FMR (trunk fat:limb fat), which ranged from 0.03 to 0.05, was greater than that previously reported for total fruit and vegetable intake and similar to that reported for adherence to a Mediterranean diet, for which associations were reported as 0.004 (95% CI: −0.07, 0.07) per serving of fruit, 0.03 (95% CI: −0.09, 0.04) per serving of vegetables, and 0.06 (95% CI −0.09, −0.02) per unit of Mediterranean diet score with the FMR defined as the ratio of leg-to-trunk fat mass (32). This outcome may suggest that flavonoid-rich fruit and vegetable intake may be more beneficial in reducing adiposity than is total fruit and vegetable intake. In our cross-sectional, multivariate-adjusted models, the mean ± SE regression coefficient for the FMR per quintile of anthocyanin intake (−0.008 ± 0.004) was 8 times greater than the valued per quintile of total fruit and vegetable intake (−0.001 ± 0.004).

Our results, with the use of objective data from DXA scans, provide further insight into the role that a higher dietary flavonoid intake may play in reducing adiposity and add mechanistic insights to previous longitudinal studies that have examined flavonoid intake and changes in weight and BMI (1, 2). Our findings for the fat mass distribution reflect our previous findings for the weight change in 3 large prospective cohorts, whereby we reported that higher habitual intakes of a range of flavonoid subclasses (with the exception of flavones and flavanones) were inversely associated with weight gain with the strongest associations observed for intakes of anthocyanins and polymers (2).

Habitual tea consumption (a mean of 434 mL/d for 10 y) has previously been associated with lower body fat and waist circumference compared with drinking no tea (33). A meta-analyses of short-term trials showed that catechins from green tea with caffeine decreased BMI, body weight, and waist circumference more than did caffeine alone although the effect sizes were modest (BMI: −0.6; body weight: −1.4 kg; and waist circumference: −1.9 cm) (34). Longer-term randomized controlled trials are needed to examine the magnitude of the effect of dietary flavonoids on body weight and fat distribution and to examine the additional benefit of flavonoid-rich foods for other weight-management regimes. Our finding that flavan-3-ols were associated with fat mass and fat mass distribution independent of genetic factors is supported by mechanistic research that showed a role for EGCG in weight maintenance (3–7). Although we showed no association with total tea intake and fat mass in this cohort, a subset analysis in participants for whom data were available on green tea intake (*n* = 1194) showed that the mean ± SE FMI was 0.26 ± 0.13 kg/m^2^ lower (*P* = 0.04), and the CFMI was 0.52 ± 0.22 kg/m^2^ lower (*P* = 0.02) in consumers than in nonconsumers.

The magnitude of the associations that we observed between intakes of the flavonoid subclasses and the FMR highlight the potential public health importance of these findings. For example, in our multivariate-adjusted model, the mean ± SE coefficient for each quintile of proanthocyanidin intake (−0.013 ± 0.004) was 2.6 times greater than the value per quintile of energy intake (0.005 ± 0.006), 1.9 times greater than the value per quartile of sugar-sweetened beverage intake (0.007 ± 0.004), and 3.3 times greater than the values of each category of physical activity (−0.004 ± 0.006). These associations were related to a mean difference in intake of 329 mg, which was equivalent to 3.2 medium apples (100 g each) or 2.8 small bars of dark chocolate (50 g each). For the other subclasses, our associations were shown with differences in intake of 78 mg flavanones [1.1 medium oranges (160 g each)] and 41 mg anthocyanins (0.3 cups blackberries (45 g) or 0.2 cups blueberries (25 g)], and 436 mg flavan-3-ols [1.5 mugs of green tea (260 g each)]. These intakes of foods can be readily incorporated into the diet, which highlights that a simple dietary change has the potential to have a great impact on weight management.

Strengths of the current study include the large sample of well-characterized participants, the measurement of all major flavonoid subclasses, and the use of a co-twin, case-control model that allowed us to examine associations independently of genetic confounding (35). The FFQ that was used in the current study has been shown to reflect habitual dietary intake and has the ability to rank participants according to intakes of flavonoid-rich foods (18, 36). Although questions have been raised about the validity and value of a self-reported dietary assessment, the measurement error in exposure assessments were likely to attenuate true associations toward the null (37–40). In addition, the use of DXA technology provided us with valid and reliable direct measures of total fat mass and regional fat mass. There were also limitations that included the cross-sectional study design, which meant that we were unable to infer causation from these findings, and because of the novel and exploratory nature of the analyses, a number of hypothesis-driven comparisons were made. Measurement errors are inevitable in FFQ estimates of dietary intake. However, we believe that we analyzed our data appropriately by ranking participants into quintiles of intake and presented our results cautiously. Findings from a recent controlled-feeding study indicated that self-report dietary data contain very low levels of underreporting for many foods and dietary constituents and not all foods are misreported to the same extent (41). Fruit and vegetable estimations from self-reported dietary assessments have been shown to correlate well with urinary flavonoid concentrations, thereby showing that FFQs can discriminate between intakes of fruit and vegetables in the normal everyday range (36). Residual or unmeasured confounding was possible despite our detailed adjustment of a range of dietary and lifestyle confounder variables. Physical activity was self-reported, and there was likely to be a large variation in the amount and intensity of activity in each of our reported categories. Because our cohort consisted only of women, we could not extrapolate our results to men. Finally, although our FFQ captured the main sources of flavonoids that are present in the habitual diet, the FFQ may not have captured all sources, and the flavonoid contents of food are variable and depend on the geographical origin, season, and processing methods.

In conclusion, our data suggest a protective role for a number of flavonoid subclasses, including anthocyanins, flavan-3-ols, and flavonols, on the distribution of fat mass independent of shared genetic and common environmental factors. We show greater associations between the FMR and flavonoid subclass intake than for physical activity and intakes of energy and sugar-sweetened beverages, which are well-known contributors to fat mass. We also show that these associations are both independent, and the effect sizes with the FMR are markedly greater than for total fruit and vegetable intake and fiber intake. Furthermore, these associations are shown with dietary achievable intakes of flavonoids, thereby making them relevant for public health recommendations to reduce body fat. Our results suggest that dietary flavonoids may contribute to a healthier fat mass profile and, thus, merit further investigation in randomized controlled trials.

## Footnotes

5 Abbreviations used: CFMI, central fat mass index; DXA, dual-energy X-ray absorptiometry; EGCG, epigallocatechin gallate; FFQ, food-frequency questionnaire; FMI, fat mass index; FMR, fat mass ratio.
